# Quantification of lincomycin resistance genes associated with lincomycin residues in waters and soils adjacent to representative swine farms in China

**DOI:** 10.3389/fmicb.2013.00364

**Published:** 2013-12-03

**Authors:** Liang Li, Jian Sun, Baotao Liu, Donghao Zhao, Jun Ma, Hui Deng, Xue Li, Fengyang Hu, Xiaoping Liao, Yahong Liu

**Affiliations:** Department of Veterinary Pharmacology and Toxicology, National Reference Laboratory of Veterinary Drugs Residues, College of Veterinary Medicine, South China Agricultural UniversityGuangzhou, China

**Keywords:** culture-independent method, agriculture field, swine farm, *lnu*(F), *erm*(A), *erm*(B)

## Abstract

Lincomycin is commonly used on swine farms for growth promotion as well as disease treatment and control. Consequently, lincomycin may accumulate in the environment adjacent to the swine farms in many ways, thereby influencing antibiotic resistance in the environment. Levels of lincomycin-resistance genes and lincomycin residues in water and soil samples collected from multiple sites near wastewater discharge areas were investigated in this study. Sixteen lincomycin-resistance and *16S rRNA* genes were detected using real-time PCR. Three genes, *lnu*(F), *erm*(A), and *erm*(B), were detected in all water and soil samples except control samples. Lincomycin residues were determined by rapid resolution liquid chromatography-tandem mass spectrometry, with concentrations detected as high as 9.29 ng/mL in water and 0.97 ng/g in soil. A gradual reduction in the levels of lincomycin-resistance genes and lincomycin residues in the waters and soils were detected from multiple sites along the path of wastewater discharging to the surrounding environment from the swine farms. Significant correlations were found between levels of lincomycin-resistance genes in paired water and soil samples (*r* = 0.885, *p* = 0.019), and between lincomycin-resistance genes and lincomycin residues (*r* = 0.975, *p* < 0.01). This study emphasized the potential risk of dissemination of lincomycin-resistance genes such as *lnu*(F), *erm*(A), and *erm*(B), associated with lincomycin residues in surrounding environments adjacent to swine farms.

## Introduction

Antibiotics are commonly used in large confined animal feeding operations (CAFOs) worldwide to promote animal growth and treat animal diseases. Many antibiotics are poorly absorbed in the gut of treated animals and therefore, up to 75% of them can be excreted in an unmetabolized form via feces and urine, allowing antibiotics to persist and accumulate in water and soil (Kumar et al., 2005). It was previously reported that a high concentration (7820 ng/mL) of lincomycin could be detected in liquid manure of swine following administration in feed. In liquid manure, ~84% of the lincomycin was in the dissolved phase, and 16% was associated with the solid components of the manure (Kuchta and Cessna, 2009b). Additionally, lincomycin could be detected in lagoon manure over a period of 5 months when applied as an amendment to agricultural land. When livestock manure from CAFOs is used as liquid fertilizer, antibiotics may transport to surface and ground water, as well as soil, and act as a reservoir (Hornish et al., 1987; Kuchta and Cessna, 2009b; Kwon, 2011). Therefore, lincomycin is one of the antibiotics that could easily accumulate in the environment adjacent to CAFOs (Hu et al., 2010).

A previous study reported that application of animal manure could lead to the potential spread of antibiotic resistance to environmental bacteria by lateral gene transfer (Ghosh and Lapara, 2007). Moreover, antibiotic resistance genes (ARGs) may transfer between pathogens and non-pathogens under selection pressure in the environment (Kruse and Sorum, 1994). Tetracycline-resistance genes, plasmid-mediated quinolone resistance genes, and chloramphenicol resistance genes were reportedly found in wastewater and soil adjacent to swine farms in China (Wu et al., 2010; Li et al., 2012, 2013). There are three known mechanisms responsible for resistance to lincomycin (Schmitz et al., 2000; Lozano et al., 2012): the *23S rRNA* methylases [encoded by *erm*(A), *erm*(B), *erm*(C), *erm*(TR)]; O-nucleotidyltransferases [encoded by *lnu*(A), *lnu*(B), *lnu*(C), *lnu*(D)] and lincomycin export mediated by efflux [*vga*(A), *vga*(B), *vga*(C), *vga*(D), *vga*(E), *lsa*(A), *lsa*(B), *lsa*(C)]. *vga*(A), *erm*(B), and *erm*(A) were detected in swine farm manure and waste treatment systems and *vga*(A)-positive pathogens were recovered from swine and swine farmers (Chen et al., 2010; Mendes et al., 2011; Zhu et al., 2013). However, few investigations have searched for lincomycin-resistance genes (especially for *erm*, *lnu*, and *vga*) in the environment of swine farms.

China is the largest producer and consumer of antibiotics in the world, and almost half of them are used in livestock industries (Hvistendahl, 2012). Also, swine manure water has been used as fertilizers of fish ponds, which when connected with surrounding waterways will promote the growth of photosynthetic organisms in China. Once swine manure water was discharged to the environment lincomycin-resistance genes and lincomycin residues most likely occur in waters and soils, and subsequently could form a selection pressure to environmental ecology.

**Figure 1:**
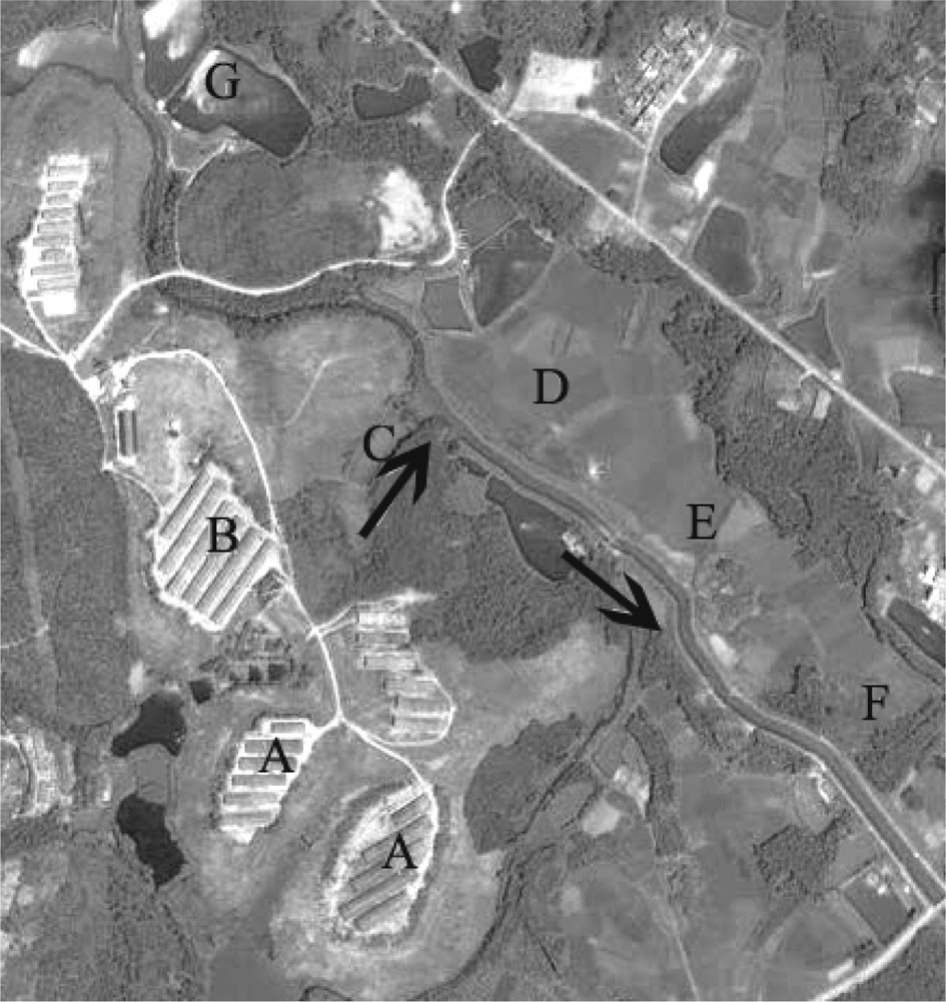
**Geographical map of the swine farm and its surrounding environment**. The black arrow indicates the direction of effluent of swine manure water. Water and soil samples were collected from sites A to G. A, farrowing pen; B, nursery house; C, fish pond; D–F, agriculture fields; G, reservoir.

The aim of this study was to quantify lincomycin-resistance genes in relation to lincomycin residues in the surrounding environment (soils and waters), especially agricultural fields adjacent to swine farms. In addition, the association between the lincomycin residues and the development of lincomycin resistance was also determined. Water and soil samples were collected from multiple sites along the path of wastewater discharged to the environment from the swine farm (Figure 1), and a culture-independent method was used to investigate the levels of lincomycin-resistance genes.

## Materials and methods

### Sampling

A total of 14 water and soil samples were collected in October 2012 from a swine farm located in Guangdong, China, with an animal density of 10,000 market hogs or more each year. This swine farm is representative of farms that are disposing swine manure to the environment. Water from swine manure was collected in trenches, and then discharged into a fish pond that connects to surrounding waterways through the ditches. Meanwhile, the water in waterways was subsequently applied to surrounding agriculture fields through irrigation (Figure 1). Methods of sample collection and preparation were described in detail by Li et al. (2012). Specifically, water samples (about 1 L for each site) were collected from six sites along the path of wastewater discharged to the environment from the swine farm (Figure 1): the farrowing pen (site A), the nursery house (site B), the fish pond (site C), and surrounding agriculture fields (sites D–F) (designated as A-w, B-w, C-w, D-w, E-w, and F-w for water samples). Soil samples (about 200 g for each site) were collected from the bottom of the trench which was used to discharge of wastewater adjacent to the water collection sites (site A and B), water-sediments of the fish pond (site C), and surrounding agriculture fields (sites D–F) (designated as A-s, B-s, C-s, D-s, E-s, and F-s for soil samples). In addition, control specimens were collected from a reservoir (site G) water (G-w) and surrounding agriculture fields soils (G-s), which received no animal wastes (thus presumably no antibiotics) upstream from the swine farm. For each site, four replicates taken from four different locations were pooled to form one composite sample. The samples were immediately kept in a cooler box during transportation and stored at –80°C before DNA extraction and quantitative analysis of lincomycin residues.

### DNA extraction

DNA extraction was carried out by a culture-independent method described previously (Li et al., 2012). Total DNA from water (about 200 mL) and soil samples (about 0.25 g) were extracted with the Power Water DNA Kit (MO BIO Laboratories Inc., Carlsbad, CA, USA) and Power Soil DNA Kit (MO BIO) according to the manufacturer's instructions, respectively.

### PCR and qPCR assays

PCR assays were used to detect the presence of 16 lincomycin resistance genes [i.e., *erm*(A), *erm*(B), *erm*(C), *erm*(TR), *lnu*(A), *lnu*(B), *lnu*(C), *lnu*(D), *lnu*(F), *lsa*(A), *lsa*(B), *lsa*(C), *vga*(A), *vga*(C), *vga*(D), and *vga*(E)] in all of the environmental and control samples. Primers for these genes were either reported or newly designed (Table A1} of the Appendix). To ensure reproducibility, PCR for lincomycin-resistance genes and 16SrRNA genes was performed in triplicate using a thermal cycler (iQ5; Bio-Rad, Hercules, CA) and SYBR^®^ Premix *Ex Taq*™ in parallel with a negative control in every run. All PCR products were directly sequenced, and the results were compared against those in the GenBank nucleotide database (http://www.ncbi.nlm.nih.gov/blast/). Primers used for real-time PCR for detecting lincomycin-resistance genes were the same as those used in the qualitative PCR, while primers for the *16S rRNA* genes were reported previously (Li et al., 2012). Each reaction was performed in a 25 μL volume containing 12.5 μL of SYBR Premix *Ex Taq*, 0.5 μL of each primer, 9.5 μ L of ddH_2_O and 2 μ L of template, with the following conditions: denaturing at 94°C for 5 min, followed by 40 cycles at 94°C for 1 min, 60°C for 1 min, and 72°C for 1 min, with a final extension at 72°C for 5 min. The melt curve was read every 1°C, from 60 to 95°C toward the end of PCR reactions. All agents were supplied by TaKaRa (TaKaRa Bio, Dalian, China). The PCR efficiencies were examined to test inhibition. The *R*^2^-values were more than 0.9 for all calibration curves. In order to minimize variance caused by differential extraction and analytical efficiencies, and differences to the background bacterial abundance, the level of each lincomycin resistance gene was normalized with the *16S rRNA* copy number using the method recommended previously (Livak and Schmittgen, 2001).

### Quantification of lincomycin

Extraction and quantitative analysis of lincomycin in water, soil, and control samples were performed according to methods described previously (Peru et al., 2006; Hu et al., 2010). Lincomycin residues were detected by rapid resolution liquid chromatography-tandem mass spectrometry (RRLC-MS/MS), comprising an Agilent liquid chromatography 1200 series RRLC system (Agilent Technologies, Palo Alto, CA, USA) coupled to an API 4000 triple quadrupole mass spectrometer (Applied Biosystems, Foster, CA, USA) with the software of Analyst 1.5 The analytical column was a 2.1 mm ID × 150 mm, 1.8 μm Zorbax SB-Aq (Agilent Technologies, Atlanta, GA, USA). The recoveries for lincomycin based on matrix-matched calibration were 90.1% in water samples and 78.4% in soil samples. The quantification limits were 10 pg/mL for water and 0.1 ng/g for soil, respectively.

### Statistics

Statistical evaluation of the data was conducted by SPSS version 17.0. The homogeneity of values was assessed via a One-Way analysis of variance (ANOVA) test. A two-tailed Pearson's bivariate correlation analysis was used to compare levels of lincomycin resistance genes in paired water and soil samples and to correlate levels of lincomycin-resistance genes with concentrations of lincomycin residues. The relative quantification of lincomycin resistance genes *lnu*(F), *erm*(A), and *erm*(B), presented in all of the water and soil samples, were conducted by correlation analysis.

## Results

### Concentration of lincomycin in waters and soils

**Figure 2:**
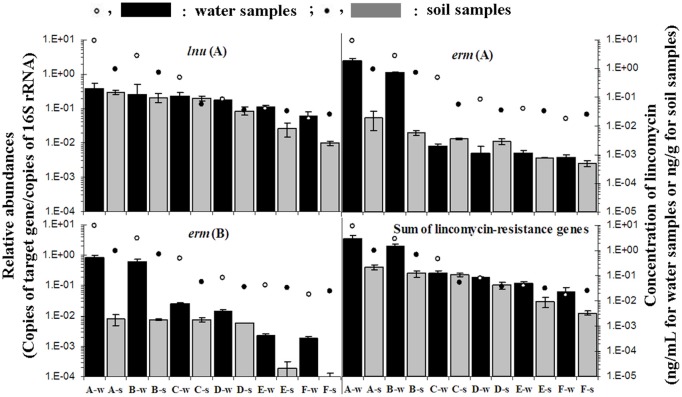
**Relative abundance of lincomycin-resistance genes: *lnu*(F), *erm*(A), *erm*(B), and the sum of the nine lincomycin resistance genes [*lnu*(F), *erm*(A), *erm*(B), *lnu*(A), *lnu*(D), *vga*(C), *vga*(E), *vga*(A), and *vga*(D)] and concentration of lincomycin**. Black bars and blank symbols indicate a relative abundance of lincomycin resistance genes and concentration of lincomycin in water samples, respectively; Gray bars and symbols indicate relative abundance of lincomycin resistance genes and concentration of lincomycin in soil samples, respectively. Error bars represent the standard deviation.

Lincomycin residues were commonly detected in all water samples (ranged from 0.018 to 9.29 ng/mL) and soil samples (ranging from 0.024 to 0.97 ng/g) from sites A to F (Figure 2). In contrast, concentrations of lincomycin were below the detection limit in all control samples. Among both water and soil samples, the highest concentrations of lincomycin were observed in the A-w sample (9.29 ng/mL) and the A-s sample (0.97 ng/g), respectively, and then declined substantially downstream of the site A. Lincomycin residues detected in water samples were higher than those in soil samples.

### Occurrence and levels of lincomycin resistance genes in waters and soils

Prevalence of lincomycin resistance genes in each sample tested are presented in Table A2. Of the 16 lincomycin resistance genes investigated, *lnu*(F), *erm*(A), and *erm*(B) were detected in all water and soil samples except the control samples. The *vga*(C) was found in all water samples; while *vga*(E) was found only in two water samples (A-w and B-w). *vga*(A) and *vga*(D) were detected in two water (A-s and B-s) and four (A-s, B-s, C-s, and D-s) soil samples, respectively. No novel sequences were observed.

Relative quantification of nine lincomycin resistance genes is shown in Figure 2 and Table A3. The level of individual lincomycin resistance genes varied in samples from site to site when present. A gradual reduction in relative quantification of lincomycin resistance genes in water and soil samples was detected from site A to F. Moreover, levels of lincomycin resistance genes in water were higher than those in soil samples from each site.

### Correlation analysis

Significant positive correlation was observed for the concentration of lincomycin residues between paired water and soil samples (*r* = 0.925, *p* = 0.008). Additionally, quantification of lincomycin resistance genes *lnu*(F) (*r* = 0.981, *p* = 0.001), *erm*(A) (*r* = 0.958, *p* = 0.003), and total lincomycin-resistance genes [*lnu*(F), *erm*(A), *erm*(B), *lnu*(A), *lnu*(D), *vga*(C), *vga*(E), *vga*(A), and *vga*(D)] (*r* = 0.885, *p* = 0.019) in water samples was significantly correlated with soil samples, except *erm*(B) (*r* = 0.626, *p* = 0.184). Significant correlations were exhibited between the level of *erm*(A) (*r* = 0.982, *p* < 0.01), *erm*(B) (*r* = 0.919, *p* < 0.01), the sum of the nine lincomycin resistance genes (*r* = 0.975, *p* < 0.01) and lincomycin residues. The level of *vga*(C) was significantly correlated with lincomycin residues in water samples (*r* = 0.999, *p* < 0.01). However, moderately significant correlations between lincomycin residue and relative quantification of *lnu*(F) (*r* = 0.705, *p* = 0.01) (Table 1) were noted.

**Table 1:** **Correlation analysis of lincomycin-resistance genes in paired water and soil samples as well as correlation between lincomycin-resistance genes and lincomycin residues**.

	***lnu*(F)**	***erm*(A)**	***erm*(B)**	**Sum**^a^****
Relative quantification	0.981	0.958	0.626	0.885
	*0.001*	*0.003*	*0.184*	*0.019*
Lincomycin residues	0.705	0.982	0.919	0.975
	*0.01*	*<0.01*	*<0.01*	*<0.01*

In each cell, the top value indicates the Pearson correlation coefficient (r), and the bottom value in italics indicates the p-value. Bold values indicate statistical significance (p < 0.05).

a Sum of nine lincomycin-resistance genes [lnu(F), erm(A), erm(B), lnu(A), lnu(D), vga(C), vga(E), vga(A) and vga(D)].

## Discussion

Since swine manure water was discharged from the site A, it was not surprising that the highest concentrations were observed in the A-w samples and the A-s samples. Concentrations of lincomycin in the A-w sample and B-w, C-w, D-w, E-w samples detected in this study were similar to previous studies of liquid swine manure and ground water from manure-amended cropland, respectively (Kuchta and Cessna, 2009a; Kuchta et al., 2009). However, lincomycin residues in soil samples were higher than that in manure-amended soil reported previously (Kuchta et al., 2009). Lincomycin residues declined substantially downstream of site A probably as a result of dilution. The accumulation of lincomycin in the environmental water and soil samples is likely attributed to the use of lincomycin as feed additives in the facility. Our findings indicated that the swine manure water was a lincomycin reservoir. Lincomycin residues in D-s, E-s, and F-s samples maintained at a relatively constant level which suggests that they are close to the detection limit of the residue detection method used.

A total of nine lincomycin resistance genes were detected in this study. The genes *erm*(A), *erm*(B), and *lnu*(F) were widespread. They were found in almost all of the water and soil samples. This is similar to a previous report where a high level of the *erm* gene was present in typical swine manures (Chen et al., 2010). Although water and soil bacterial communities are different, *erm*(A) and *erm*(B) could spread widely due to the discharge of swine manure water containing ARGs in the environment. In this study, it was noteworthy that levels of *vga*(A), and *lnu*(A) in swine manure water were 10-fold higher than previous reports in soil and water samples, respectively (Zhu et al., 2013). *lnu*(D), *vga*(C), *vga*(D), and *vga*(E) were sporadically detected in environmental samples from sites close to site A. Contrary to previous studies (Zhu et al., 2013), *vga*(B) was not detected in any sample in our study. To our knowledge, this is the first report of the presence of *lnu*(F), *lnu*(D), *vga*(C), *vga*(D), and *vga*(E) genes in surrounding environments adjacent to swine farms in China. Lincosamide O-nucleotidyltransferases encoded by *lnu*(A), *lnu*(D), *lnu*(F), and ATP-binding cassette transporters encoded by *vga*(A), *vga*(C), *vga*(D), and *vga*(E) could also be transferred by plasmids and transposons; thus it is posing a potential dissemination risk (Petinaki et al., 2008; Kadlec et al., 2010; Schwendener and Perreten, 2011).

Similar to the levels of plasmid-mediated quinolone resistance genes in environmental samples, we found that the levels of lincomycin resistance genes in water were higher than those in soil samples at each site (Li et al., 2012). Significant correlation between paired water and soil samples with regard to the relative levels of lincomycin resistance genes and lincomycin residues was found. Lincomycin resistance genes and lincomycin residues were not detected in control samples (site G). This indicates that the swine manure water is the reservoir of these contaminants. Similar reservoirs of ARGs and antibiotics are probably common in China and other countries (Zhu et al., 2013). Previous studies suggested that there was the exchange of ARGs between environmental bacterial and clinical pathogens, which might pose health risks to nearby residents exposed to contaminated field soil, fish pond water, and waterway water during farming (Forsberg et al., 2012; Li et al., 2012).

Considering that high levels of *vga*(C) and *vga*(D) were only detected in soil samples and the level of *vga*(C) was significantly correlated with lincomycin residues, it may be concluded that environmental quantification of lincomycin resistance genes was not only influenced by a dilution effect of contamination but also by a selective effect from lincomycin residues in the environment. Previous studies indicated that antibiotic residues in the environment (soil and water) could affect the selection and dissemination of resistance genes, and promote or inhibit ecological functions (Ghosh and Lapara, 2007; Naslund et al., 2008; Schauss et al., 2009; Ding and He, 2010). Therefore, long-term use of lincomycin, coupled with its slow degradation in soil and water (Kuchta et al., 2009; Williams and McLain, 2012) could potentially lead to the selection of resistant bacteria species and the transfer of ARGs located in transferable elements (Looft et al., 2012). This hypothesis will be investigated in future work.

### Conflict of interest statement

The authors declare that the research was conducted in the absence of any commercial or financial relationships that could be construed as a potential conflict of interest.

## Figures and Tables

**Table A2:**
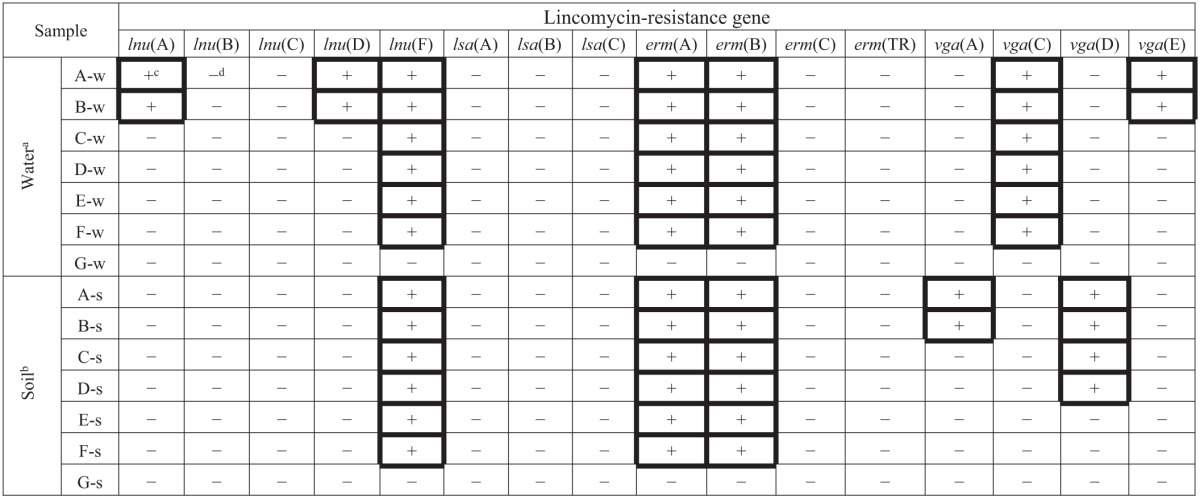
**PCR detection results of lincomycin-resistance genes in total DNA**.

^a^Water samples were collected from 6 sites along the effluent of swine manure water.

^b^Soil samples were collected from soils adjacent to the effluent of swine manure water.

^c^+: positive result was presented with a shadow box.

^d^-: negative result.

**Table A3:**
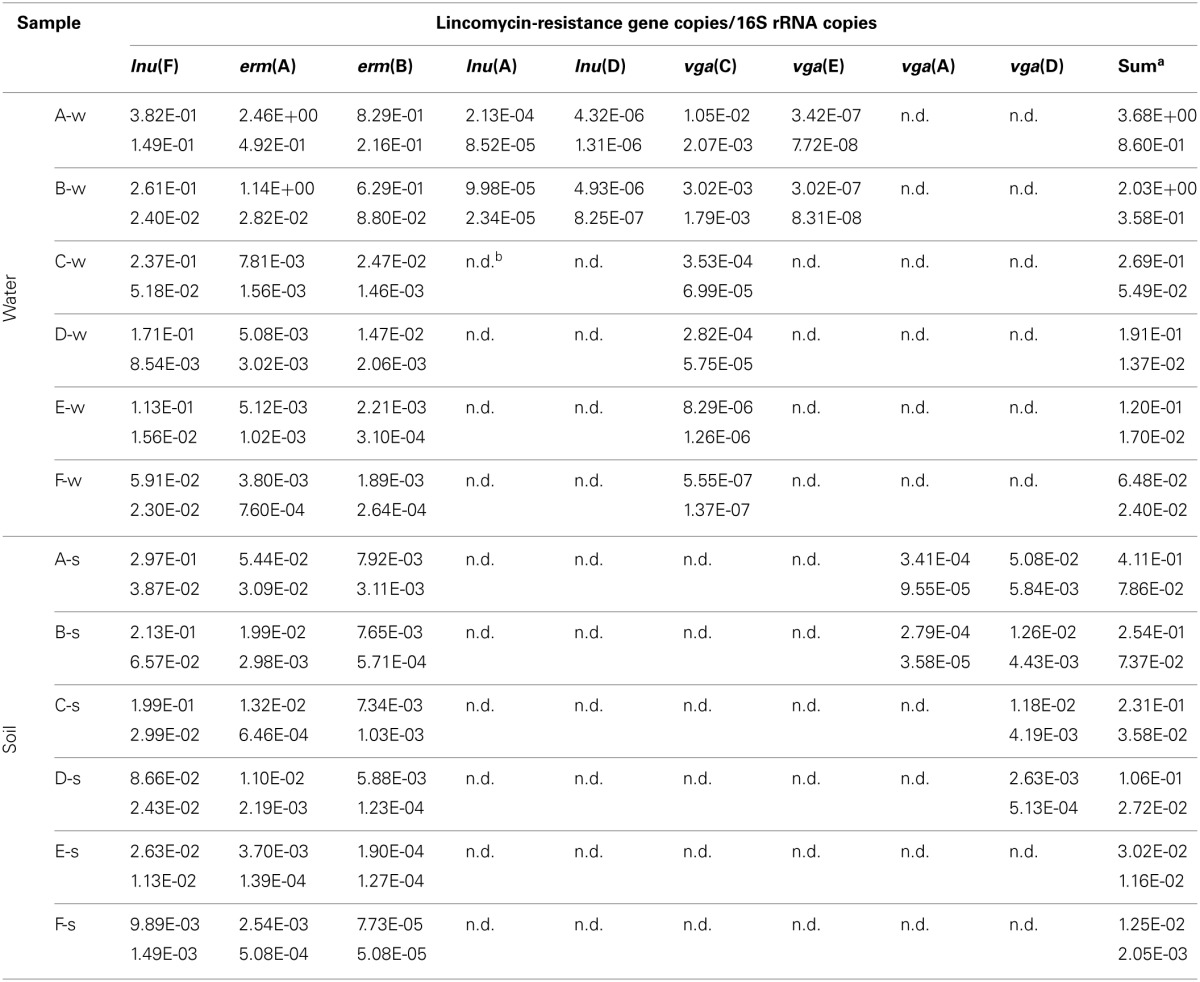
**Relative quantification of lincomycin-resistance genes in water and soil samples, normalized to corresponding 16S rRNA copies**.

In each cell, the top value indicates the means of three replicates determined by qPCR, and the bottom value indicates the standard deviation.

^a^Sum of nine lincomycin-resistance genes [lnu(F), erm(A), erm(B), lnu(A), lnu(D), vga(C), vga(E), vga(A), and vga(D)].

^b^n.d.: not detected.

## Appendix

**Table A1:** **PCR (QPCR) primers and conditions used in this study**.

**Gene**	**Oligonucleotide sequence (5′-3′)**	**Product size (bp)**	**Temperature (°C)**	**References**
*lnu*(A)	GGTGGCTGGGGGGTAGATGTATTAACTGG	323	57	Lina et al., 1999
	GCTTCTTTTGAAATACATGGTATTTTTCGA			
*lnu*(B)	CCTACCTATTGTTTGTGGAA	925	54	Bozdogan et al., 1999
	ATAACGTTACTCTCCTATTTC			
*lnu*(C)	AATTTGCAATAGATGCGGAGA	1100	55	Luthje and Schwarz, 2007
	TCATGTGCATTTTCATCA			
*lnu*(D)	ACGGAGGGATCACATGGTAA	475	56	Haenni et al., 2011
	TCTCTCGCATAATAACCTTACGTC			
*lnu*(F)	CACCATGCTTCAGCAGAAAATGATC	1200	55	De Graef et al., 2007
	TTACTTGTTGTGCGGCGTC			
*lsa*(A)	CGCTCCAGCTGTATGAGAACTGC	1200	55	Singh et al., 2002
	TCAAGCGATTGACTTCTTTTTTG			
*lsa*(B)	TGATATTGTCTCTTGGAAGG	1100	56	Kehrenberg et al., 2005
	AATGAACGCTTGCAGAAGGA			
*lsa*(C)	GGCTATGTAAAACCTGTATTTG	429	55	Malbruny et al., 2011
	ACTGACAATTTTTCTTCCGT			
*erm*(A)	GTTCAAGAACAATCAATACAGAG	421	52	This study
	GGATCAGGAAAAGGACATTTTAC			
*erm*(B)	GAAAAGGTACTCAACCAAATA	639	52	This study
	AGTAACGGTACTTAAATTGTTTAC			
*erm*(C)	TCAAAACATAATATAGATAAA	642	47	Sutcliffe et al., 1996
	TAACTGCTAAATTTGTTATAATCG			
*erm*(TR)	ACAGAAAAACCCCGAAAAATACG	679	54	Tait-Kamradt et al., 2000
	TTGGATAATTTATCAAGATCAG			
*vga*(A)	AGTGGTGGTGAAGTAACACG	659	55	Werner et al., 2001
	CTTGTCTCCTCCGCGAATAC			
*vga*(C)	TAGCAGACGAACCGACGACC	863	51.5	This study
	TTCACCACCGCTTAGCACAT			
*vga*(D)	CAACTGGAGCGAGCTGTTA	201	55	Jung et al., 2010
	GACAGCCGGATAATCTTTTG			
*vga*(E)	GAAATATGGGAAATAGAAGATGG	512	52	Schwendener and Perreten, 2011
	TGATTCTCTAACCACTCTTC			
*16S rRNA*	GGTAGTCYAYGCMSTAAACG	263	62	Bach et al., 2002
	GACARCCATGCASCACCTG			
